# MicroRNA399 is a long-distance signal for the regulation of plant phosphate homeostasis

**DOI:** 10.1111/j.1365-313X.2007.03363.x

**Published:** 2008-03

**Authors:** Bikram Datt Pant, Anja Buhtz, Julia Kehr, Wolf-Rüdiger Scheible

**Affiliations:** Max-Planck Institute for Molecular Plant Physiology Science Park Golm, Am Mühlenberg 1, 14476 Potsdam, Germany

**Keywords:** microRNA, phosphate homeostasis, long-distance signaling, nutrient, Arabidopsis, Brassica

## Abstract

The presence of microRNA species in plant phloem sap suggests potential signaling roles by long-distance regulation of gene expression. Proof for such a role for a phloem-mobile microRNA is lacking. Here we show that phosphate (Pi) starvation-induced microRNA399 (miR399) is present in the phloem sap of two diverse plant species, rapeseed and pumpkin, and levels are strongly and specifically increased in phloem sap during Pi deprivation. By performing micro-grafting experiments using Arabidopsis, we further show that chimeric plants constitutively over-expressing miR399 in the shoot accumulate mature miR399 species to very high levels in their wild-type roots, while corresponding primary transcripts are virtually absent in roots, demonstrating shoot-to-root transport. The chimeric plants exhibit (i) down-regulation of the miR399 target transcript (*PHO2*), which encodes a critical component for maintenance of Pi homeostasis, in the wild-type root, and (ii) Pi accumulation in the shoot, which is the phenotype of *pho2* mutants, miR399 over-expressers or chimeric plants with a genetic knock-out of *PHO2* in the root. Hence the transported miR399 molecules retain biological activity. This is a demonstration of systemic control of a biological process, i.e. maintenance of plant Pi homeostasis, by a phloem-mobile microRNA.

## Introduction

Inorganic phosphate (Pi) is one of the most limiting mineral nutrients for plant growth (Bieleski, 1973; Poirier and Bucher, 2002; Marschner, 1995). The levels in the soil are often low, much of the Pi is covalently or non-covalently bound, and mobility is restricted. Strategies of plants to cope with and to improve Pi acquisition during Pi limitation include increasing the root/shoot ratio (Lynch, 1995), alterations in root architecture (López-Bucio *et al.*, 2002; Williamson *et al.*, 2001), production of lateral roots into Pi-rich patches in the soil (Robinson, 1994), proliferation of long root hairs (Bates and Lynch, 2001), increasing high-affinity Pi uptake capacities (Mudge *et al.*, 2002; Smith *et al.*, 2003), establishing symbiotic interactions with mycorrhizal fungi (Harrison, 1999), production and root secretion of organic acids to solubilize Pi in the soil and apoplast (Raghothama, 1999), and induction of phosphatases (del Pozo *et al.*, 1999; Li *et al.*, 2002) and RNases (Bariola *et al.*, 1994) that mobilize organically bound phosphate inside and outside the plant. Changes in metabolism allow re-mobilization of Pi in the plant, including a general decrease in the levels of P-containing intermediates and co-factors such as nucleotides (Zrenner *et al.*, 2006), or replacement of phospholipids by sulfo- and galactolipids (Dörmann and Benning, 2002; Härtel *et al.*, 2000).

Correct allocation of the available Pi in the plant, i.e. maintenance of Pi homeostasis by supplying growing organs and tissues such as young leaves and inflorescences with the spare resources taken up by roots, is also important for plant productivity and survival during Pi limitation. The existence of mutants with altered levels and distribution of Pi (*pho1*, *pho2* and *pup1*; see Delhaize and Randall, 1995; Poirier *et al.*, 1991; Trull and Deikman, 1998) suggests that plants regulate the long-distance transport and allocation of Pi between shoot and root. *PHO1* encodes the founder member of a novel transporter family (Hamburger *et al.*, 2002) and probably is involved in Pi transport into the xylem (Wang *et al.*, 2004), and *PHO2* encodes an E2 ubiquitin-conjugating enzyme (Aung *et al.*, 2006; Bari *et al.*, 2006). The PHO2 E2 conjugase is a major component for the maintenance of Pi homeostasis in Arabidopsis. *pho2* mutants grown in Pi-replete conditions display continuous induction of a subset of phosphate starvation-induced (PSI) genes, including some encoding phosphate transporters (Aung *et al.*, 2006; Bari *et al.*, 2006), and accumulate Pi to high levels (three- to fivefold increased) in shoots but not roots (Bari *et al.*, 2006; Delhaize and Randall, 1995).

PHO2 expression is post-transcriptionally regulated by microRNA399 (miR399). miR399 acts through transcript cleavage (Allen *et al.*, 2005) and probably also translational repression (Bari *et al.*, 2006). Consistently, over-expression of miR399 leads to high shoot Pi levels, thus phenocopying *pho2* mutants (Aung *et al.*, 2006; Bari *et al.*, 2006; Fujii *et al.*, 2005). The *PHO2* gene harbors five binding sites complementary to miR399 in its 5′ UTR approximately 200–400 nucleotides upstream of the start codon, and miR399-dependent *PHO2* transcript cleavage at these sites has been experimentally verified (Allen *et al.*, 2005). Six miR399 species (a–f) that arise from five primary transcripts (PTs) have been identified in Arabidopsis (http://microrna.sanger.ac.uk). miR399d is by far the most prominent one during Pi limitation, based on the abundance of its primary transcript (Bari *et al.*, 2006). The levels of mature miR399 and the five miR399 PTs are highly and specifically increased during Pi limitation (Bari *et al.*, 2006; Fujii *et al.*, 2005), suggesting inhibition of PHO2 under these conditions. Consistently, removal of the miR399 binding sites results in high and stable *PHO2* transcript levels in Pi-limited plants (Fujii *et al.*, 2005). Micrografting experiments revealed that a *pho2* mutant root genotype is sufficient to result in shoot Pi accumulation (Bari *et al.*, 2006). On the other hand, *MIR399* genes, and especially *MIR399d*, display particularly strong induction in shoots (as compared to roots) during Pi limitation, with the shoot miR399d PT level exceeding the root *PHO2* transcript level (Bari *et al.*, 2006). This led us to hypothesize that miR399 itself might act as a long-distance Pi starvation signal in Arabidopsis.

Long-distance signaling in plants is known to be important for the regulation of several processes including leaf development, flowering, pathogen defense and resource allocation (Jaeger and Wigge, 2007; Khamis *et al.*, 1990; Palauqui *et al.*, 1997; Voinnet *et al.*, 1998; for review, see Lough and Lucas, 2006). The phloem translocation stream of higher plants contains a multitude of small molecules and macromolecules, including proteins, mRNA and small RNAs (Lough and Lucas, 2006; Ruiz-Medrano *et al.*, 2001). Several microRNAs have also been detected in the phloem sap of pumpkin (Yoo *et al.*, 2004) and oilseed rape (Buhtz *et al.*). However, unlike siRNA or mRNA species (Haywood *et al.*, 2005; Ruiz-Medrano *et al.*, 1999; Voinnet *et al.*, 1998; Yoo *et al.*, 2004), microRNAs have not been functionally implicated in plant long-distance signaling. Here we present data that identify miR399 as a phloem-mobile long-distance signal for regulation of Pi homeostasis in Arabidopsis.

## Results and discussion

### Presence and Pi-specific induction of miR399 in phloem sap

To demonstrate the presence of miR399 and its Pi-status-dependent changes in phloem sap, we grew rapeseed (*Brassica napus*) and pumpkin (*Cucurbita maxima*) plants under abundant and limiting Pi conditions, and collected phloem sap for the determination of microRNA levels. Doing this type of experiment with Arabidopsis is impracticable because of (i) major difficulties associated with the collection of sufficiently large quantities of phloem sap from this small plant species, (ii) problems with RNase contamination and/or wounding-inducible RNase activity (Köck *et al.*, 2004; LeBrasseur *et al.*, 2002) in phloem sap exudates collected by decapitation or incision methods, making isolation and quantification of RNA impossible (A.B. and J.K., unpublished observations), and (iii) contamination of the sap with content (e.g. RNA) from other cells. Rapeseed plants are a useful alternative to Arabidopsis due to the close genetic relatedness of the two species, as well as their similar phloem sap composition, as judged by their protein profiles (J.K., unpublished data). In addition, phloem sap exudation from incised rapeseed inflorescence stems is quite strong, thus enabling rapid sampling and minimization of problems involving contaminating/induced RNase activity.

**Figure 1:**
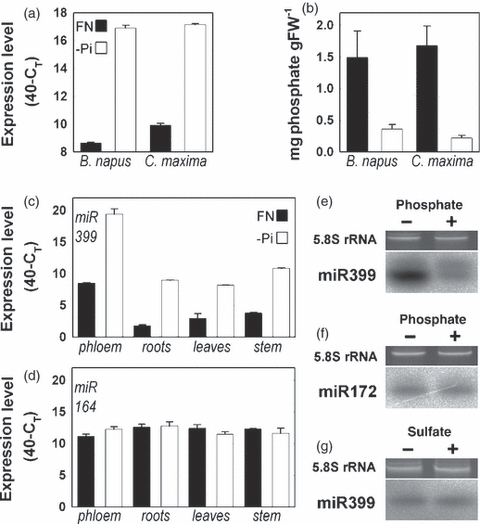
Mature miR399 levels in phloem sap change with phosphate status. (a) Levels of mature miR399 in phloem saps of *Brassica napus* and *Curcurbita maxima* grown under full nutrient (FN, black bars) and Pi-limited (white bars) conditions. Data (mean ± SE, *n* = 3) depict 40 − *C*_T_ values, i.e. the cycle number when PCR ends (the threshold cycle number of the amplicon). Note the logarithmic scale (log_(1 + *E*)_, where *E* is the PCR efficiency) of the *y* axis. miR399 cDNA produced from 10 ng total RNA was included in each assay. (b) Pi levels in leaves of FN-grown and Pi-limited *Brassica napus* and *Curcurbita maxima* plants. Data are the mean±SE (*n* = 4 or 5). (c, d) Levels of mature miR399 and mature miR164 in phloem sap, roots, leaves and stems of *Brassica napus* grown under full nutrient (black bars) and Pi-limited (white bars) conditions, as determined by quantitative real-time PCR. Data are depicted as in (a). miR399 or miR164 cDNA produced from 20 ng total RNA was included in each assay. Data are the mean ± standard deviation of two biological replicates (with two technical replicates for each), each pooled from 4–10 plants in independent experiments. (e–g) RNA gel blot signals for miR399 (e, g) and miR172 (f) in phloem-sap of *Brassica napus* plants grown with 0.5 mm (+) or without (−) phosphate (e,f) or sulfate (g). An aliquot (10 μg) of total RNA isolated from pooled samples (*n* = 4–10) was loaded in each lane. 5.8S rRNA is shown as a loading control.

Mature miR399 was detected by quantitative real-time PCR and RNA gel blotting in rapeseed and pumpkin phloem sap (Figure 1), but miR399 precursors or primary transcripts were not detectable (results not shown). Mature miR399 levels increased very strongly in the phloem sap of Pi-limited plants (a difference of 7–10 quantitative real-time PCR *C*_T_ value units, equivalent to a >100-fold change) (Figure 1a–c,e). The increase was even stronger and the abundance higher in phloem sap than in leaves, for example (Figure 1c), suggesting efficient transport of miR399 into the sap and/or strong induction of miR399 expression in phloem companion cells. The increase/accumulation of miR399 in phloem sap or other plant organs such as roots was also specific, as levels for other microRNA species, i.e. miR164 (Figure 1d) and miR172 (Figure 1f), did not change during Pi limitation. These results demonstrate that miR399 is present and highly abundant in the phloem, and indicate that the increase during Pi limitation is not related to decreased phloem mass flow. They also underscore the conserved Pi-responsiveness of miR399 in distantly related plant species (Bari *et al.*, 2006; Chiou *et al.*, 2006).

We further investigated the Pi specificity of this response by investigating miR399 levels in phloem sap of plants starved of sulfate. RNA gel blotting showed that mature miR399 abundance was unaffected by sulfate deprivation in phloem sap (Figure 1g) and leaves and roots (results not shown) of rapeseed plants. This is in accordance with previous results showing that miR399 primary transcripts are unaffected by limitation of sulfate, nitrogen or carbohydrates (Bari *et al.*, 2006). It was also demonstrated that mature miR399 is unaffected by nitrogen (Bari *et al.*, 2006) or potassium limitation (Fujii *et al.*, 2005). Taken together, these results show that the responsiveness of miR399 and its primary transcripts is Pi-specific in all plant organs/samples, including phloem sap.

### Micrografting reveals shoot-to-root transport and biological activity of shoot-derived miR399 in roots

**Figure 2:**
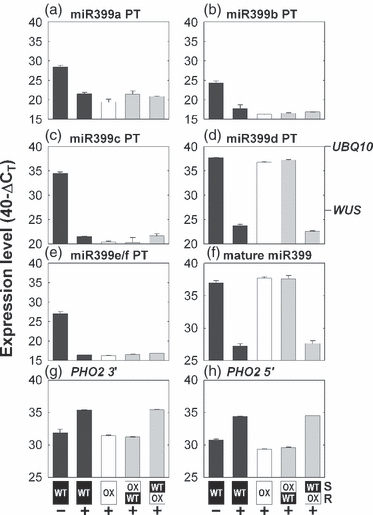
Quantitative real-time PCR expression levels of miR399 and *PHO2* in shoots. Levels of (a–e) the five miR399 primary transcripts (PT), (f) mature miR399, and (g, h) *PHO2* in shoots of WT (black bars), miR399 OX (white bars), and micrografted chimeric plants (gray bars). Shoot (S) and root (R) genotypes of the chimeric plants are depicted below the graphs. Plants were grown with Pi (+) or were Pi-starved (−). Expression levels are given on a logarithmic scale expressed as 40 – Δ*C*_T_, where Δ*C*_T_ is the difference in quantitative real-time PCR threshold cycle number (*C*_T_ value) between the studied gene and the reference gene *UBQ10* (*At4g05320*); 40 therefore equals the expression level of *UBQ10* (the number 40 was chosen because the PCR run stops after 40 cycles). The fold difference in expression is 2^ΔΔ*C*T^ when the PCR efficiency is 2 (e.g. an ordinate value of 30 represents 1000-fold lower expression than a value of 40). The results are the mean ± standard deviation for two biological replicates each pooled from four or five shoots in independent experiments.

**Figure 3:**
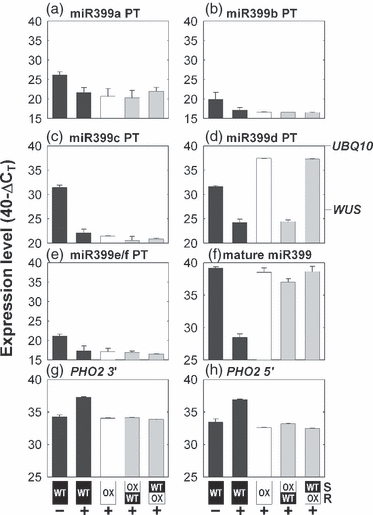
Quantitative real-time PCR expression levels of miR399 and *PHO2* in roots. Levels of (a–e) the five miR399 primary transcripts (PT), (f) mature miR399, and (g,h) *PHO2* in shoots of WT (black bars), miR399 OX (white bars), and micrografted chimeric plants (gray bars). Shoot (S) and root (R) genotypes of the chimeric plants are depicted below the graphs. Plants were grown with Pi (+) or were Pi-starved (−). Expression levels are given as described in the legend to Figure 2. The results in (a–e), (g) and (h) are the mean ± standard deviation of two biological replicates pooled from 3–6 seedling roots in independent experiments, and the results in (f) are the mean ± standard deviation of three biological replicates pooled from 2–6 roots in independent experiments. Two technical replicates were measured for each biological replicate.

We next investigated whether miR399 produced in the shoot can be detected and consequently alter expression of its target gene (*PHO2*) in the root. For this purpose, we used micro-grafting (Turnbull *et al.*, 2002) to generate chimeric Arabidopsis plants that over-express miR399 in either shoots or roots. Six miR399 species (a–f) that arise from five primary transcripts have been identified in Arabidopsis. miR399d is the most prominent one during Pi limitation, based on the abundance of its primary transcript (Bari *et al.*, 2006) (Figures 2 and 3). Transgenic plants strongly over-expressing *miR399d* (OX) under control of the 35S promoter were previously shown to have approximately 10-fold lower levels of the target *PHO2* transcript (Bari *et al.*, 2006), and to accumulate Pi in leaves to levels as high as in *pho2* mutants or in micro-grafted chimeric plants with a *pho2* root and a wild-type (WT) shoot scion (Bari *et al.*, 2006).

**Figure 4:**
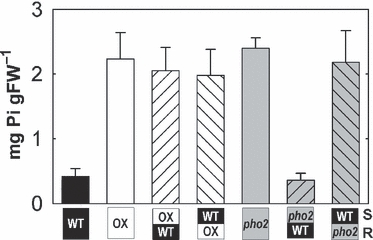
Leaf phosphate levels in chimeric plants. Phosphate levels in leaves of 5-week-old, hydroponically grown WT (black bars), miR399 OX (white bars), *pho2* mutant (gray bars) and reciprocally grafted chimeras (hatched bars). Shoot (S) and root (R) genotypes of the chimeric plants are depicted below the graph. Data are mean values ± SE (*n* = 4 or 5).

As expected, the primary transcripts of miR399d (miR399d PT) and mature miR399 were strongly increased (threshold fluorescence was reached 12–13 cycles earlier during quantitative real-time PCR, equivalent to a >1000-fold increase) in root and shoot material from OX plants compared to the same material from WT plants (Figures 2 and 3 and Table S1). The levels of miR399d PT and mature miR399 in OX shoots matched the high levels found in Pi-starved WT shoots (Figure 2d,f), but the levels of the other miR399 primary transcripts (a, b, c, e/f) remained at barely detectable or undetectable levels, as in Pi-replete WT (see Table S1; note that in Figures 2 and 3 values for 40-deltaCT of less than approximately 30 correspond to transcript copy numbers of far less than 1 per cell in our quantitative real-time PCR system (Czechowski *et al.*, 2004; also see Experimental Procedures)). In Figures 2 and 3, values for 40 −Δ*C*_T_ of less than approximately 30 correspond to transcript copy numbers of much less than 1 per cell in our quantitative real-time PCR system (see Experimental procedures). The level of *PHO2* transcript, measured using two quantitative real-time PCR primer pairs amplifying near the 3′ and 5′ ends of the *PHO2* coding sequence (CDS), was approximately 10-fold decreased in OX plants (equivalent to 3 or 4 quantitative real-time PCR cycles) (Figure 2g,h and Table S1), and the leaf Pi level was four- to fivefold increased (Figure 4) as previously reported (Bari *et al.*, 2006).

When shoot material from chimeras with an OX shoot and WT root (OX/WT) was investigated, it showed the same behavior as OX shoots of non-chimeric plants with respect to the levels of miR399 PTs, mature miR399 and *PHO2* transcript (Figure 2). The root material of the same OX/WT chimeras exhibited WT-like miR399 PT levels (Figure 3a–e and Table S1), which were all below the expression level of some of the rarest transcription factor transcripts known (e.g. *WUSCHEL*, *At2g17950*), equivalent to an abundance of approximately 1 copy per 100 cells or less for each miR399 PT (see Czechowski *et al.*, 2004). In contrast, the same WT roots had very high, OX-like, mature miR399 levels (Figure 3f), equivalent to a copy number of > 500 per cell, which is similar to that of one of the most abundant plant transcripts, *UBQ10* (see Czechowski *et al.*, 2005) and OX-like low *PHO2* transcript level (Figure 3g,h). The shoot material had high Pi levels in the leaves, similar to those of genotypes in which PHO2 is inhibited in roots (Figure 4). We have previously shown by micro-grafting of *pho2* and WT that decreased PHO2 in the shoot alone does not lead to increased Pi in the shoot (Bari *et al.*, 2006).

**Figure 5:**
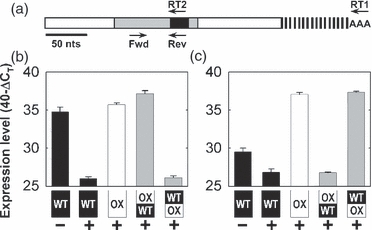
Absence of miR399d precursor fragments in roots of OX/WT chimeras. (a) Depiction of miR399d primary transcript structure. miR399 is shown in black, the miR399 precursor in gray, and the residual parts of the known transcript in white. The hatched part towards the 3′ end is of unknown size. RT1 is the oligo(dT) primer, RT2 is the reverse transcription primer used for cDNA synthesis for the quantitative real-time PCR measurements shown in (b) and (c), together with quantitative real-time PCR primers Fwd and Rev. (b, c) Expression levels of the quantitative real-time PCR amplicon in (b) shoots and (c) roots. For additional information, see legends to Figures 2 and 3.

A theoretical explanation for the virtually absent miR399 PTs and the simultaneous massive accumulation of mature miR399 in roots of OX/WT chimeras could be highly efficient cleavage of miR399 PTs into shorter fragments that escape oligo(dT)-primed reverse transcription and subsequent quantitative real-time PCR detection. Although there is no indication of highly efficient cleavage in roots of OX plants or WT/OX chimeras (i.e. the level of miR399 PTs mirrors the mature miR399 level), we analyzed whether there is an increase of corresponding fragments by reverse-transcribing total RNA with a primer binding to the 3′ region of the miR399d precursor sequence (Figure 5) (see Experimental procedures). However, there was no evidence for accumulation of such fragments in the roots of the OX/WT chimeras, as the level was as low as in Pi-replete WT. Similarly, the miR399 complementary duplex strand (miR399*) could not be detected in roots of OX/WT chimeras or roots of Pi-replete WT, but was clearly present in Pi-limited WT roots and miR399d PT over-expresser roots (results not shown). These results further suggest that the *MIR399* genes are truly inactive in the roots of the OX/WT chimeras. The explanation for the massive accumulation of mature miR399 found in roots of OX/WT chimeras therefore must be transport of miR399 molecules from the OX scion to the WT root, where they exert their biological function, i.e. suppress *PHO2*. Co-localization of miR399 and the *PHO2* target transcript in the root vascular cylinder has been reported previously (Aung *et al.*, 2006), and further supports their interaction *in vivo*. The inactivity of the *MIR399* genes in the roots of OX/WT chimeras also excludes the possibility that another phloem-mobile molecular species (e.g. a hormone or metabolite) acts as an intermediate signal to trigger miR399 synthesis.

A situation similar to that in the OX/WT chimera was also observed in Pi-deprived WT plants. During Pi starvation, levels of the miR399 PT rise in WT roots, but only to levels that are approximately 1% of the level of mature miR399 (Figure 3 and Figure S1a), while miR399 PT levels in WT shoots equal or exceed the mature miR399 level (Figure 2 and Figure S1a). This result indicates that miR399 phloem transport is relevant in the normal biological situation. In addition, given the relatively long half-life time of mature miR399 (approximately 12 h, Figure S1b) (Bari *et al.*, 2006), we estimated that phloem transport rates are sufficiently high to achieve and maintain very high (i.e. *PHO*-inhibitory) levels of mature miR399 in roots that do not express miR399 PTs within a physiological response time of 2–3 h (see Appendix S1). In summary, Pi deprivation in the shoot can be rapidly signaled to the roots through miR399. Subsequent inhibition of PHO2 by miR399 in the root will lead to increased Pi uptake and transport to the shoot (Aung *et al.*, 2006; Bari *et al.*, 2006; Delhaize and Randall, 1995). This elegant mechanism is capable of maintaining shoot Pi homeostasis and optimizing shoot growth during Pi limitation.

### No evidence for root-to-shoot transport of miR399

Reciprocal chimeras (WT/OX) that over-express miR399d PT and mature miR399 in roots were also investigated. The root material (Figure 3) resembled that of normal OX plants, while the shoot material (Figure 2) resembled WT material, with low miR399d PT and low mature miR399. This indicates that root-to-shoot transport of miR399, which would occur via the xylem, does not take place or occurs to a negligible extent. Unlike water or ions, the transport of larger molecules, including miR399, is not circulatory between shoot and root (Lough and Lucas, 2006). Accordingly, miR399 was not detectable in xylem sap from oilseed rape plants grown under normal or phosphate-deficient conditions (Buhtz *et al.,* in press). The level of *PHO2* transcript in the shoots of WT/OX chimeras was high, resembling Pi-replete WT shoots (Figure 2g,h). Leaf Pi levels were also high (Figure 4), resembling genotypes in which the level of *PHO2* transcript is low in roots (Bari *et al.*, 2006).

Taken together, these results pinpoint the mature miR399 as a phloem-mobile molecule that increases in concentration in phloem sap during Pi limitation, and that can be transported between plant organs and exert its biological role at its destination. Hence, miR399 fulfills the criteria for a long-distance signaling molecule. The existence of multiple microRNA species in phloem sap of diverse plant species (Buhtz *et al.,* in press; Yoo *et al.*, 2004) indicates that this might only be one example of systemic control of a biological process by a microRNA.

Despite the demonstration that miR399 is transported in the phloem conduit, and thereby plays a central role in the maintenance of whole-plant Pi homeostasis, several questions remain to be addressed with respect to the transport mechanism. These include the mechanism and proteins required for miR399 entry into and exit from the sieve tubes, and the requirement and nature of RNA binding proteins to allow miR399 phloem transport, such as the 27 kDa phloem small RNA binding protein CmPSRP1 (Yoo *et al.*, 2004) or the 16 kDa phloem protein 16 (CmPP16) (Xoconostle-Cázares *et al.*, 1999) in *Cucurbita maxima*. With respect to translocation in the phloem, the current data indicate that miR399, as already suggested for the whole miRNA population (Yoo *et al.*, 2004), travels as a single-stranded molecule, as, in contrast to the miR399 sense strand, the level of the miR399* strand does not show any increase under phosphate starvation (Buhtz *et al.,* in press). miR399 is efficiently translocated from Pi-replete OX shoots to Pi-replete WT roots. This suggests that the components of miR399 phloem uptake, translocation and exit are either constitutively present or induced during Pi limitation in a miR399-dependent manner.

Long-distance signaling in biological systems was put in place when multi-cellular organisms evolved. In this regard, it is interesting to note that miR399 and its target *PHO2* gene are found in higher multi-cellular plants only, including rice, poplar and *Medicago*, but not in single-celled algae (Bari *et al.*, 2006). The Pi-dependence of miR399 expression is strongly conserved between dicots and monocots, as is the target gene with respect to gene structure, encoded protein sequence, and the number and positions of miR399 binding sites in its 5′ UTR (Bari *et al.*, 2006). This suggests co-evolution and conservation of the molecular components required for Pi homeostasis in higher multi-cellular plants. Long-distance transport of miR399 further cements this view.

## Experimental procedures

### Growth of rapeseed and pumpkin, and phloem sap sampling

Rapeseed (*Brassica napus* cv. Drakkar) and pumpkin (*Cucurbita maxima*) plants were grown in 18 cm pots containing a low-Pi soil mix in a phytotron at 25°C (day)/20°C (night), with a 16 h light period at an intensity of 550 μE. Plants were divided into two sets; one set was supplied with a full nutrient solution containing sufficient (3 mm) Pi, and the other one was supplied with the same nutrient solution free of Pi. Rapeseed plants were grown until flowering (7–8 weeks) and phloem sap was then collected by incising inflorescence stems (Giavalisco *et al.*, 2006). Phloem sap from pumpkin plants was collected by stem incision 3 weeks after germination. After discarding the first droplets, the exuded phloem sap was collected into four volumes of ice-cold Trizol reagent (Invitrogen, http://www.invitrogen.com/).

Hydroponically grown rapeseed plants were germinated on filter paper for 1 week before transferring them to a hydroponics system containing nutrient medium (Buhtz *et al.,* in press) for 7–8 weeks. Nutrient solutions were changed after 3 weeks, and subsequently renewed once a week. Phosphate and sulfur starvation were initiated after 3 weeks by changing to medium containing no phosphate or sulfur, respectively, and phloem sap was collected from inflorescence stems as described above.

### Micrografting of Arabidopsis seedlings

Micrografting of 6-day-old seedlings grown on sterile vertical agar plates was performed as described previously (Turnbull *et al.*, 2002). Seeds of the miR399d over-expressers, wild-type (ecotype Col-0) and *pho2* mutant were surface-sterilized, and stratified at 4°C for 3 days. The seed were then laid onto sterile half-strength MS agar (0.7%) plates. The plates were kept vertically in constant light (approximately 120 μE) at 20°C for 3 days in an Arabidopsis growth chamber (Percival Scientific Inc., http://www.percival-scientific.com/), and then kept at 28°C under an 8 h photoperiod (60 μE) for another 3 days. The seedlings were grafted using silicon tubing (0.3 mm internal diameter) as the collar, and grafted seedlings were kept on identical sterile agar plates and under the same growth conditions for another 6–8 days until the graft junction had healed. Successfully grafted plantlets (approximately 14 days old) were transferred to a hydroponic culture system with half-strength nutrient solution containing 1.5 mm or no Pi (Scheible *et al.*, 2004), and grown for another 3 weeks before harvesting of leaf and root material into liquid nitrogen. This material was used for analysis of gene expression and Pi content. Special care was taken to avoid any plants with adventitious roots.

### Pi measurements

Pi levels were measured using a colorimetric micromethod (Itaya and Ui, 1966). Discs were taken from leaves of the same age using a 6 mm cork borer, and individually ground in 50 μl distilled water using plastic rods (Sarstedt, http://www.sarstedt.com) in 96-well plates (Greiner Bio-One, http://www.greinerbioone.com). Extracts were diluted with water to 125 μl, and then the microtiter plate was centrifuged at 3000 ***g***for 4 min. Aliquots (10 μl) of the supernatant were mixed with 15 μl water, and 100 μl 1 m HCl and 100 μl color reagent (one volume of 4.2% (NH_4_)_6_Mo_7_O_24_·H_2_O in 5 N HCl, three volumes of 0.2% malachite green dye in water) were added. The plate was incubated at room temperature for 15 min before adding 100 μl 1.5% Tween-20. The absorbance at 660 nm was measured another 15 min later. The Pi concentration in the samples was determined against a calibration curve.

### RNA isolation, cDNA preparation, and quantitative real-time PCR

RNA isolation of total RNA from *Brassica napus* and *Curcurbita maxima* was achieved using Trizol reagent, and isolation of RNA from roots and shoots of micrografted Arabidopsis plants was performed using RNeasy mini kits (Qiagen; http://www.qiagen.com). cDNA preparation and quantitative real-time PCR were performed using the primer pairs previously described (Bari *et al.*, 2006; Czechowski *et al.*, 2004, 2005). cDNA synthesis for the detection of miR399d precursors/primary transcript fragments was primed using 5′- GGCAAATCTCCTTTGGCAGAG-3′ (RT2 in Figure 5). Typically, approximately 5–10 ng of cDNA, the amount produced from the RNA of several thousand cells (Czechowski *et al.*, 2004), was used per assay, yielding a *C*_T_ value of 16–17 for *UBQ10* (see Table S1), which is equivalent to 1–2 million single-stranded *UBQ10* template copies in the PCR reaction vessel.

For quantification of mature miR399 and miR164, total RNA was reverse-transcribed using stem-loop primers (5′-GTCGTATCCAGTGCAGGGTCCGAGGTATTCGCACTGGATACGACCGGGGCAAA-3′ and 5′-GTCGTATCCAGTGCAGGGTCCGAzGGTATTCGCACTGGAT ACGACCGCACG-3′, respectively) and MultiScribe reverse transcriptase (Applied Biosystems; http://www.appliedbiosystems.com/) (Chen *et al.*, 2005). In brief, 1 μg total RNA was mixed with 1 μl 10 mm dNTPs, 1 μl 2.5 μm miRNA-specific stem-loop RT primer and RNase-free water to a final volume of 36.5 μl, heated to 65°C for 5 min, and then chilled on ice. Then 10 μl of 5x first-strand buffer, 2 μl 0.1 m DTT, 0.5 μl RNase inhibitor and 1 μl MultiScribe reverse transcriptase were added. The reaction mixture was incubated for 30 min at 16°C, followed by 30 min at 42°C and 5 min at 85°C, and then held at 4°C until quantitative real-time PCR amplification using primers miR399fwd (5′-CGACGTGCCAAAGGAGATTTG-3′), miR164fwd (5′-CACGTGGAGAAGCAG GGCA-3′) and miRrev (5′-CCAGTGCAGGGTCCGAGGT-3′). Rather than TaqMan probes (Chen *et al.*, 2005), SYBR® Green (Applied Biosystems; http://www.appliedbiosystems.com) was used as the fluorescence dye for real-time monitoring of DNA amplification.

Primer efficiency (*E*) was determined for all amplicons using the freeware program LinRegPCR (Ramakers *et al.*, 2003). The value did not change significantly between different cDNA samples, and was always higher than 0.80.

### RNA gel blot analysis of microRNA species

Northern analysis was performed as described by Bari *et al.* (2006). Aliquots (20 μg) of total RNA were resolved on denaturing 17% polyacrylamide gels containing 7 m urea in 0.5x Tris-Borate/EDTA. RNA was blotted to positively charged nylon membranes (Biodyne B, Pall Europe Ltd; http://www.pall.com/) using a semi-dry transfer cell (Bio-Rad; http://www.bio-rad.com/) and auto-crosslinked at 0.12 mJ in a Stratalinker 1800 (Stratagene; http://www.stratagene.com/). The membrane was hybridized at 42°C with ^32^P end-labeled probes (5′-TTACAGGGCAAATCTCCTTTGGCA-3′ and 5′-ATGCAGCATCATCAAGATTCT-3′) complementary to miR399 and miR172, respectively, and visualized using a phosphorimager BAS-1800 II (Fuji Photo Film Co. Ltd; http://www.fujifilm.com/).

Materials not commercially available and used in the experiments reported will be made available for non-profit research on request.

## Supplementary material

The following supplementary material is available for this article online:

Figure S1 Expression levels of mature miR399 and miR399d primary transcripts in Arabidopsis wild-type during Pi limitation and re-addition.

Table S1 Quantitative real-time RT-PCR threshold cycle values.

Appendix S1 Estimation of the time required to establish high mature miR399 levels by shoot-to-root phloem transport in roots that do not express miR399 PTs.

This material is available as part of the online article from http://www.blackwell-synergy.com
