# Supplementary figures and images for: MicroRNA399 is a long-distance signal for the regulation of plant phosphate homeostasis
Source: Plant J. 2008 Mar;53(5):731–8. doi: 10.1111/j.1365-313X.2007.03363.x (PMC2268993; doi:10.1111/j.1365-313X.2007.03363.x)

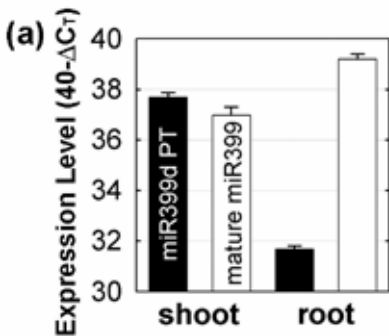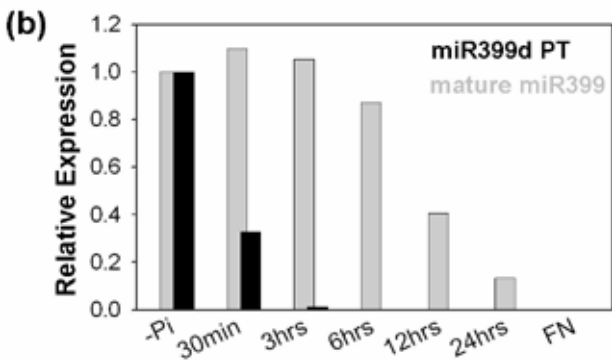

Supplement: Figure S1 — Expression levels of mature miR399 and miR399d primary transcripts in Arabidopsis wild-type during Pi limitation and re-addition. [file tpj0053-0731-SD1.pdf]
