# Supplementary material for: MicroRNA399 is a long-distance signal for the regulation of plant phosphate homeostasis
Source: Plant J. 2008 Mar;53(5):731–8. doi: 10.1111/j.1365-313X.2007.03363.x (PMC2268993; doi:10.1111/j.1365-313X.2007.03363.x)
Supplement: Table S1 — Quantitative real-time RT-PCR threshold cycle values. [file tpj0053-0731-SD2.doc]

**Table S1.** Quantitative real-time RT-PCR threshold cycle values.

| **Amplicon** | **WT (-Pi)** | **WT (FN)** | **miR399d OX** | **399dOX Shoot**  **WT Root** | **WT Shoot**  **399dOX Root** |
| --- | --- | --- | --- | --- | --- |
| **Shoots** | | | | | |
| 399aPT | 27.97 ± 0.39 | 34.88 ± 0.38 | 36.84 ± 0.79 | 35.1 ± 0.77 | 35.99 ± 0.01 |
| 399bPT | 32.12 ± 0.57 | 38.66 ± 0.95 | 40 (n.d.) | 40 (n.d.) | 40 (n.d.) |
| 399cPT | 21.89 ± 0.27 | 34.89 ± 0.07 | 35.87 ± 0.18 | 36.25 ± 1.04 | 35.2 ± 0.39 |
| 399dPT | 18.7 ± 0.08 | 32.64 ± 0.23 | 19.46 ± 0.07 | 19.33 ± 0.07 | 34.3 ± 0.09 |
| 399efPT | 29.31 ± 0.52 | 40 (n.d.) | 40 (n.d.) | 40 (n.d.) | 40 (n.d.) |
| 399 mature | 19.41 ± 0.33 | 29.15 ± 0.14 | 18.51 ± 0.20 | 18.91 ± 0.27 | 29.23 ± 0.15 |
| PHO2 3' | 24.51 ± 0.55 | 21.03 ± 0.05 | 24.82 ± 0.11 | 25.3 ± 0.02 | 21.42 ± 0.01 |
| PHO2 5' | 25.64 ± 0.17 | 21.99 ± 0.04 | 26.87 ± 0.05 | 26.93 ± 0.07 | 22.36 ± 0.02 |
| UBQ10 | 16.38 ± 0.11 | 16.4 ± 0.05 | 16.22 ± 0.08 | 16.53 ± 0.07 | 16.86 ± 0.13 |
| PDF2 | 21.33 ± 0.14 | 21.52 ± 0.17 | 21.55 ± 0.03 | 22.04 ± 0.04 | 21.79 ± 0.06 |
| **Roots** | | | | | |
| 399aPT | 30.99 ± 0.74 | 35.43 ± 1.25 | 35.97 ± 2.02 | 36.33 ± 1.89 | 34.6 ± 1.03 |
| 399bPT | 37.33 ± 1.78 | 40 (n.d.) | 40 (n.d.) | 40 (n.d.) | 40 (n.d.) |
| 399cPT | 25.78 ± 0.51 | 34.96 ± 0.70 | 35.16 ± 0.04 | 36.08 ± 0.87 | 35.7 ± 0.18 |
| 399dPT | 25.54 ± 0.13 | 32.86 ± 0.62 | 19.19 ± 0.02 | 32.15 ± 0.32 | 19.27 ± 0.03 |
| 399efPT | 36.02 ± 0.44 | 40 (n.d.) | 40 (n.d.) | 40 (n.d.) | 40 (n.d.) |
| 399 mature | 18.03 ± 0.19 | 28.6 ± 0.53 | 18.11 ± 0.71 | 19.57 ± 0.71 | 17.92 ± 0.79 |
| PHO2 3' | 22.94 ± 0.28 | 19.79 ± 0.05 | 22.57 ± 0.05 | 22.49 ± 0.05 | 22.67 ± 0.02 |
| PHO2 5' | 23.77 ± 0.51 | 20.17 ± 0.05 | 24.02 ± 0.04 | 23.41 ± 0.03 | 24.06 ± 0.04 |
| UBQ10 | 17.22 ± 0.14 | 17.12 ± 0.22 | 16.62 ± 0.12 | 16.61 ± 0.11 | 16.56 ± 0.08 |
| PDF2 | 21.66 ± 0.18 | 21.87 ± 0.35 | 20.85 ± 0.15 | 21.88 ± 0.07 | 21.30 ± 0.21 |
