# Supplementary material for: MicroRNA399 is a long-distance signal for the regulation of plant phosphate homeostasis
Source: Plant J. 2008 Mar;53(5):731–8. doi: 10.1111/j.1365-313X.2007.03363.x (PMC2268993; doi:10.1111/j.1365-313X.2007.03363.x)
Supplement: Appendix S1 — Estimation of the time required to establish high mature miR399 levels by shoot-to-root phloem transport in roots that do not express miR399 PTs. [file tpj0053-0731-SD3.doc]

**Appendix S1:** Estimation of the theoretical time required to establish high mature miR399 levels by shoot-to-root phloem transport in roots that do not express miR399 PTs.

**1. Estimation of phloem transport rate in Arabidopsis.**

(i) Photosynthetic C-assimilation rate (A): **~5µmoles CO_2_ m^-2^ h^-1^** (*A.th*. plants grown in low light; Hendrik Poorter and Joachim Fisahn, personal communications) 🡪 **~10 µmoles sucrose gFW^-1^ h^-1^** (typical specific leaf weight, SLW, for low light grown *A.th.* leaves: **~150 g m^-2^** , (W. Scheible unpublished results).

(ii) Shoot to root ratio (S/R; gFW/gFW) = ~4 (W. Scheible unpublished results) 🡪 ~20% of the sucrose produced is transported to the root 🡪 **~2.0 µmoles sucrose gFW^-1^_root_ h^-1^**

(iii) [sucrose]_phloem_ = **~500mM** (Julia Kehr & Mark Stitt, personal communications) 🡪 **~4µl phloem sap gFW^-1^_root_ h^-1^**.

**2. Estimation of miR399 concentration in *Brassica napus* phloem sap relative to leaves.**

24.5 µg of total RNA were isolated from ~500µl phloem sap and 170µg of total RNA was isolated from ~200mg leaf material of P-starved rapeseed plants. MiR399 cDNA produced from 10ng total RNA was included in each qRT-PCR reaction. The PCR with phloem sap miR399 cDNA reached threshold fluorescence (C_T_ value) 11 cycles (cf. Figure 1c) earlier than the one with leaf miR399 cDNA. According to Czechowski *et al.* (2004) this equals a >2000 fold higher miR399 template number in the phloem sap RNA sample. 10ng of phloem sap RNA equal the amount extracted from 0.20 µl phloem sap, i.e. 0.22 mg (density ~1.1g l^-1^), whereas 10ng of leaf RNA equal the amount extracted from 0.0118 mg FW_leaf_. Hence on a weight basis, **miR399 is >100-fold more abundant in phloem sap than in leaf material (mg/mg)**, or >10-fold more abundant in phloem sap than in the leaf cell cytosol (volume cell/volume cytosol = ~10).

**3. Estimation of miR399 concentration in leaves and roots of *Arabidopsis* OXShoot/WTRoot chimeras.**

(i) ~250µg of total RNA can be isolated from 100mg Pi-replete leaf material. MiR399 cDNA produced from 10ng total RNA of leaf material from OX/WT plants yielded a C_T_ value = ~19. According to Czechowski *et al.* (2004) this equals 100,000 initial miR399 cDNA template copies in the reaction vessel. 🡪 25 million copies mg FW^-1^.

(ii) In analogy to *Brassica napus* we estimate the concentration of miR399 in *Arabidopsis* phloem sap to be ~100 fold higher than in leaf material 🡪 ~2.5 billion copies µl^-1^ phloem sap🡪 according to (1) **~10 billion copies are transported per hour into 1g root material.**

(iii) ~200µg of total RNA were isolated from 100mg P-replete root material. MiR399 cDNA produced from 10ng total RNA of root material from OX/WT plants yielded a C_T_ value = ~19. According to Czechowski *et al.* (2004) this again equals 100,000 initial miR399 cDNA template copies in the reaction vessel. 🡪 **20 million copies mg FW^-1^**.

🡪 the estimated time required to transport the number of miR399 molecules found per gram root material is (20 million copies mg FW^-1^ / 10 million copies mg FW^-1^ h^-1^) **approximately 2 hours**.

🡪 the phloem transport capacity is sufficiently high to keep the root well informed about the present degree of Pi starvation (i.e. miR399 levels) in the shoot.
